# Multi-Field Coupling Dynamics Modeling of Aerostatic Spindle

**DOI:** 10.3390/mi12030251

**Published:** 2021-03-01

**Authors:** Guoda Chen, Yijie Chen

**Affiliations:** 1 State Key Laboratory of Fluid Power and Mechatronic Systems, Zhejiang University, Hangzhou 310027, China; 2 College of Mechanical Engineering, Zhejiang University of Technology, Hangzhou 310023, China; chenyj@zjut.edu.cn; 3 Key Laboratory of Special Purpose Equipment and Advanced Processing Technology, Ministry of Education and Zhejiang Province, Zhejiang University of Technology, Hangzhou 310023, China

**Keywords:** dynamics modeling, aerostatic spindle, rotor trajectory, stability, Reynolds equation

## Abstract

The aerostatic spindle in the ultra-precision machine tool shows the complex multi-field coupling dynamics behavior under working condition. The numerical investigation helps to better understand the dynamic characteristics of the aerostatic spindle and improve its structure and performance with low cost. A multi-field coupling 5-DOF dynamics model for the aerostatic spindle is proposed in this paper, which considers the interaction between the air film, spindle shaft and the motor. The restoring force method is employed to deal with the times varying air film force, the transient Reynolds equation of the aerostatic journal bearing and the aerostatic thrust bearing is solved using ADI method and Thomas method. The transient air film pressure of aerostatic bearings is obtained which clearly presents the influence induced by the tilt motion of the spindle shaft. The motion trajectory of the spindle shaft is obtained which shows different stability of the shaft under different external forces. The dynamics model shows good performance on simulating the multi-field coupling behavior of the aerostatic spindle under external force. which is quite meaningful and useful for the further research on the dynamic characteristics of the aerostatic spindle.

## 1. Introduction

The aerostatic spindle plays a key role in an ultra-precision machine tool in the nano-precision machining, which directly affects the machining quality [1,2]. Generally, the shaft of the aerostatic spindle is directly driven by a permanent magnet synchronous motor (PMSM) and supported by the aerostatic bearing, including the journal bearing and the thrust bearing [3]. 

Compared to the aerodynamic bearing, the aerostatic bearing provides higher capacity and steady force, while it contains more complex mechanism contributes to the dynamic behavior of aerostatic spindle. During the operation of the aerostatic spindle, it shows the multi-physics field coupling characteristics, in which the coupling effect of air bearing, the shaft and the motor should be considered for fully understanding its dynamics behavior [4]. However, the previous literatures commonly focused on single factor or two factors while investigating the dynamic characteristics of the aerostatic spindle [5,6,7,8]. It has been pointed out that the existence of unbalanced magnetic force (UMF) caused by rotor eccentricity at the motor can have a considerable impact on the machined surface [9,10,11], thus, the electromagnetic factor is not negligible in the dynamics model of aerostatic spindle. 

Rotor dynamics modeling is the preferred method to study the dynamic characteristics of a rotor-bearing system, by which we can get detailed information of how the rotor acts under different condition. Among the previous literature, the rotor dynamics modeling method was widely used for modeling the aerodynamic bearing-rotor system with 2-DOF (Degree of Freedom) [12,13,14,15,16], the rotor trajectory and the stability problem were analyzed. Since performance superiority of aerostatic spindle emerges in recent years, the analysis on aerostatic spindle came out frequently and have made great progress. Wang [17] implemented the rotor dynamic model of aerostatic bearing-rotor systems which applied a spherical aerostatic bearing, and the nonlinear dynamic behavior of the rotor bearing system is analyzed. Similar works were done by Zhang et al. [18], they proposed forecast orbit method to deal with the transient gas lubricated Reynold equation, and the 2D rotor trajectory under different rotor speed and imbalance mass was obtained. Two-dimensional analysis can reflect the characteristics of aerostatic bearing to some extent but not as precise as the result of the 5-DOF model. In a 5-DOF model, the tilt motion, the displacement in axial direction and the conical movement of the shaft can be acquired while the 2-DOF model cannot, and these forms of movement are also proved to have significant influence on the machined surface quality by Zhang et al. [19]. To achieve this, Li et al. [20,21] presented the 5-DOF model of an aerostatic spindle in a fly-cutting machine, in which the air pressure distribution of journal bearing and thrust bearing was calculated and the displacement of the shaft in different directions was obtained. Xu and Jiang [22,23] analyzed the 5-DOF rotor dynamics model of an aerostatic spindle, in which the stability, the unbalance response, and the forced response of the rotor-bearing system were investigated. Both Li and Xu adopted the dynamic coefficient method in their model, however it may lose the numerical accuracy when taking this method which indeed largely improved the efficiency. By contrast, the restoring force commonly used in 2-DOF models aims to obtain force directly by solving the transient Reynold equation [24,25], and it can also obtain the torque in a 5-DOF model. It can be optimized of the dynamic coefficient method, but if this measure is taken, massive extra work needs to be done. Besides, the available research on aerostatic spindle modeling fail to consider the electromagnetic factor, which has important effect on the motion error of the spindle.

In this paper, the electromagnetic factor is considered in the modeling of the aerostatic spindle, and the restoring force method is adopted. The finite difference method is adopted to discrete the transient Reynolds equation. The simulation for the motion trajectory of the spindle shaft is realized under the coupling of the restoring force of the aerostatic bearing, the UMF of PMSM and the external force on the shaft, where the shaft is regarded as a rigid rotor.

## 2. Mathematical Modeling of Aerostatic Spindle

**Figure 1:**
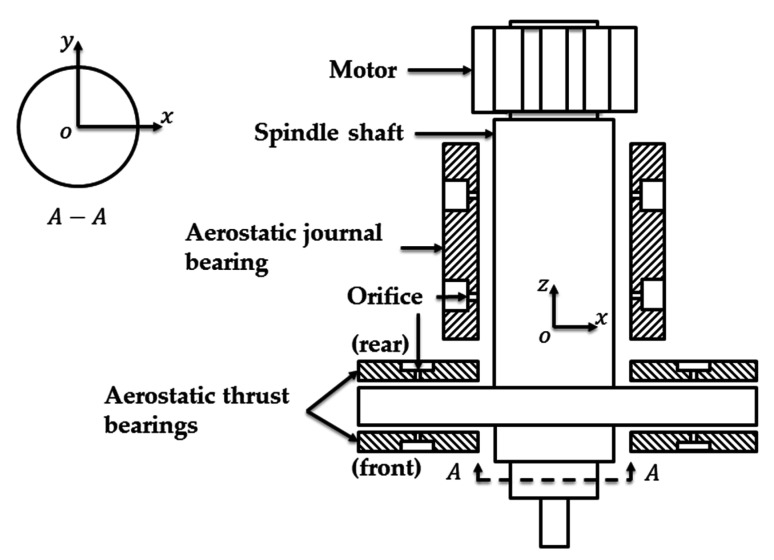
The structure diagram of the aerostatic spindle.

The typical structure diagram of the aerostatic spindle is shown in Figure 1, the spindle shaft is directly driven by the motor, and the shaft is supported by an aerostatic journal bearing and two aerostatic thrust bearing. 

Effectively modeling the dynamic behavior of the air film is quite necessary for the systematic and consistent air bearing design [26,27], thus, the modeling of the air film is of primary importance. The pressure distribution of the aerostatic bearing air film can be described by the Reynolds equation, and the Reynolds equation is obtained based on the Navier-Stokes Equations with following assumptions, the flow is isothermal, the gas viscosity is assumed to be constant, the pressure distribution in the vertical direction to the air film is assumed to be constant, the viscosity force is assumed to be much larger than inertia force, there is no velocity slip at the boundary, and the air is the ideal gas.

### 2.1. Modeling of Aerostatic Journal Bearing

The transient pressure distribution of aerostatic journal bearing can be modeled by the transient Reynolds equation in the Cartesian coordinate [28].

(1) $$\frac{\partial }{\partial x}\left(ph^{3}\frac{\partial p}{\partial x}\right)+\frac{\partial }{\partial z}\left(ph^{3}\frac{\partial p}{\partial z}\right)+\frac{12\mu P_{a}}{\rho_{a}}\rho \tilde{v}\delta_{k}=6\mu U_{0}\frac{\partial \left(ph\right)}{\partial x}+12\mu \frac{\partial \left(ph\right)}{\partial t}$$

where x is the circumferential coordinates of journal bearing, z is the axial coordinate of journal bearing, h is the air film thickness, p is the air film pressure, μ is the dynamic viscosity of the air, $U_{0}$ is the surface velocity of the shaft, $\tilde{v}$ is the flow velocity of the supply air at the orifice, t represents the time, ρ is the air density, $P_{a}$ and $\rho_{a}$ are the pressure and density of the ambient air, $\delta_{k}=1$ at the orifice and $\delta_{k}=0$ at other position.

The dimensionless transient Reynolds equation is given by

(2) $$\frac{\partial }{\partial \theta }\left(H^{3}\frac{\partial P^{2}}{\partial \theta }\right)+\frac{\partial }{\partial Z}\left(H^{3}\frac{\partial P^{2}}{\partial Z}\right)+Q\delta_{k}=\Lambda_{J}\frac{\partial \left(PH\right)}{\partial \theta }+2\Lambda_{J}\frac{\partial \left(PH\right)}{\partial \tau }$$

The dimensionless parameters are defined as follows.

$$P=\frac{p}{P_{s}},H=\frac{h}{C_{r}},\theta =\frac{x}{L_{j}},Z=\frac{z}{L_{j}},Q=\frac{24\mu R^{2}P_{a}}{C_{r}^{3}P_{s}^{2}\rho_{a}}\rho \tilde{v},\tau =\omega t,\Lambda_{J}=\frac{12\mu \omega RL_{j}}{P_{s}C_{r}^{2}}$$

where $L_{j}$ is the characteristic length of journal bearing, R is the radius of the journal bearing, $C_{r}$ is the radial clearance between the journal bearing and the shaft, $P_{s}$ is the supply pressure, $\Lambda_{J}$ is the bearing number of the journal bearing, ω is the rotating speed of the shaft.

**Figure 2:**
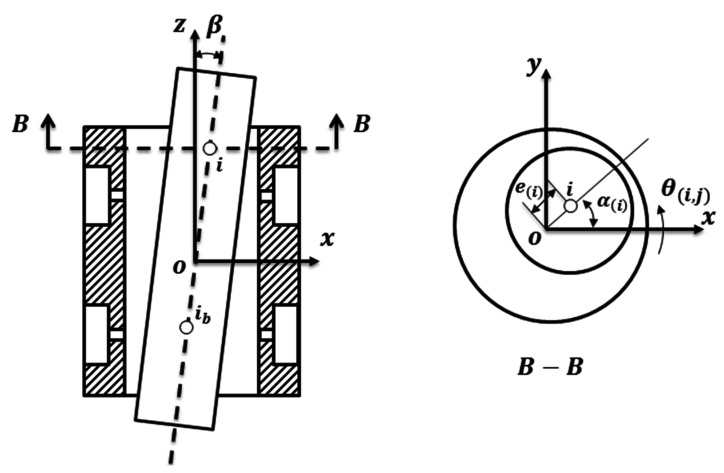
The tilt and the eccentricity of the shaft at the journal bearing.

When the eccentricity or the tilt of the shaft happens as depicted in Figure 2, the thickness of the air film changes simultaneously, and the distribution of the air film thickness in the journal bearing can be expressed as

(3) $$\{\begin{array}{c} h_{\left(i,j\right)}=C_{r}\cdot \left(1+\varepsilon_{\left(i\right)}\cdot \cos\left(\theta_{\left(i,j\right)}-\alpha_{\left(i\right)}\right)\right)\\ H_{\left(i,j\right)}=1+\varepsilon_{\left(i\right)}\cdot \cos\left(\theta_{\left(i,j\right)}-\alpha_{\left(i\right)}\right) \end{array}$$

(4) $$\varepsilon_{\left(i\right)}=\varepsilon_{ib}+\frac{\sin\left(\beta \right)\cdot \left(i-i_{b}\right)\cdot L_{s}}{M_{s}}$$

(5) $$\varepsilon_{ib}=\sqrt{{\left(e_{ibx}\right)}^{2}+{\left(e_{iby}\right)}^{2}}$$

(6) $$\alpha_{\left(i\right)}=\{\begin{array}{cc} arctg\frac{e_{iy}}{e_{ix}} & e_{ix}\geq 0\\ arctg\frac{e_{iy}}{e_{ix}}+\pi & e_{ix}<0 \end{array}$$

where $\varepsilon_{\left(i\right)}$ is the eccentricity at the node i, $\theta_{\left(i,j\right)}$ is the angle of air film at node i and j, $\alpha_{\left(i\right)}$ is the eccentric angle at the node i, $i_{b}$ is the node of the barycenter, $L_{s}$ is the axial length of the shaft, $M_{s}$ is the number of nodes that $L_{s}$ divided into, $\varepsilon_{ib}$ is the eccentricity at the node $i_{b}$, β is the tilt angle of the shaft, $e_{ibx}$ and $e_{iby}$ are the eccentricity of the node $i_{b}$ in x direction and y direction respectively, $e_{ix}$ and $e_{iy}$ are the eccentricity of the node i in x direction and y direction respectively.

The mass flow rate can be acquired based on the isothermal assumption given by Powell [29].

(7) $$\dot{m}=\phi P_{s}A_{inlet}\sqrt{\frac{2\rho_{a}}{p_{a}}}\Psi$$

(8) $$\Psi =\{\begin{array}{c} \begin{array}{cc} {\left[\frac{k}{k-1}\left(\beta_{i}{}^{\frac{2}{k}}-\beta_{i}{}^{\frac{k+1}{k}}\right)\right]}^{\frac{1}{2}} & \beta_{i}>\beta_{k} \end{array}\\ \begin{array}{cc} {\left[\frac{k}{2}{\left(\frac{2}{k+1}\right)}^{\frac{k+1}{k-1}}\right]}^{\frac{1}{2}} & \beta_{i}\leq \beta_{k} \end{array} \end{array}$$

(9) $$A_{inlet}=\{\begin{array}{c} \begin{array}{cc} \pi dC_{r} & \frac{d}{4}>C_{r} \end{array}\\ \begin{array}{cc} \frac{\pi d^{2}}{4} & \frac{d}{4}\leq C_{r} \end{array} \end{array}$$

where ϕ is the coefficient of the mass flow rate, $A_{inlet}$ is the minimum area of the flow channel, d is the diameter of the orifice, $\beta_{i}=p/P_{s}$ and $\beta_{k}={\left(2/(k+1)\right)}^{k/(k-1)}$.

**Figure 3:**
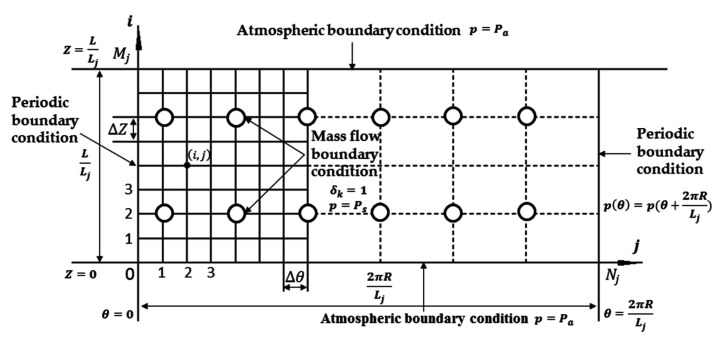
The computational domain of the journal bearing.

As shown in Figure 3, the computational domain of the journal bearing film is meshed into $\theta \left(0:N_{j}\right)$ and $Z\left(0:M_{j}\right)$, the Periodic boundary condition, the Atmospheric boundary condition and the Mass flow boundary condition are also defined.

Submitting the expression $S=P^{2}$, the central difference method is adopted to discrete the variable in the θ and Z directions. The ADI method [18] is employed to simplify the implicit equation obtained after the discretization. The ADI format of the equation can be expressed as follows,

(10) $$A_{\left(i,j\right)}S_{\left(i-1,j\right)}^{n+1}+B_{\left(i,j\right)}S_{\left(i,j\right)}^{n+1}+C_{\left(i,j\right)}S_{\left(i+1,j\right)}^{n+1}=D_{\left(i,j\right)}$$

(11) $$A_{\left(i,j\right)}=-\frac{{\left(H_{\left(i,j\right)}^{n}\right)}^{3}}{{\left(\Delta Z\right)}^{2}}+3{\left(H_{\left(i,j\right)}^{n}\right)}^{2}\frac{H_{\left(i+1,j\right)}^{n}-H_{\left(i-1,j\right)}^{n}}{{\left(2\Delta Z\right)}^{2}}$$

(12) $$B_{\left(i,j\right)}=\frac{2{\left(H_{\left(i,j\right)}^{n}\right)}^{3}}{{\left(\Delta Z\right)}^{2}}+\frac{\Lambda_{J}H_{\left(i,j\right)}^{n}}{P_{\left(i,j\right)}^{n}\Delta \tau }$$

(13) $$C_{\left(i,j\right)}=-\frac{{\left(H_{\left(i,j\right)}^{n}\right)}^{3}}{{\left(\Delta Z\right)}^{2}}-3{\left(H_{\left(i,j\right)}^{n}\right)}^{2}\frac{H_{\left(i+1,j\right)}^{n}-H_{\left(i-1,j\right)}^{n}}{{\left(2\Delta Z\right)}^{2}}$$

(14) $$\begin{array}{l} D_{\left(i,j\right)}=Q\delta_{k}+3{\left(H_{\left(i,j\right)}^{n}\right)}^{2}\frac{\left(H_{\left(i,j+1\right)}^{n}-H_{\left(i,j-1\right)}^{n}\right)\left(S_{\left(i,j+1\right)}^{n}-S_{\left(i,j-1\right)}^{n}\right)}{{\left(2\Delta \theta \right)}^{2}}+\\ {\left(H_{\left(i,j\right)}^{n}\right)}^{3}\frac{\left(S_{\left(i,j+1\right)}^{n}-2S_{\left(i,j\right)}^{n}+S_{\left(i,j-1\right)}^{n}\right)}{{\left(\Delta \theta \right)}^{2}}-\frac{\Lambda_{J}H_{\left(i,j\right)}^{n}}{2P_{\left(i,j\right)}^{n}}\frac{\left(S_{\left(i,j+1\right)}^{n}-S_{\left(i,j-1\right)}^{n}\right)}{2\Delta \theta }-\\ \Lambda_{J}P_{\left(i,j\right)}^{n}\frac{\left(H_{\left(i,j+1\right)}^{n}-H_{\left(i,j-1\right)}^{n}\right)}{2\Delta \theta }+\frac{\Lambda_{J}H_{\left(i,j\right)}^{n}S_{\left(i,j\right)}^{n}}{P_{\left(i,j\right)}^{n}\Delta \tau }-2\Lambda_{J}P_{\left(i,j\right)}^{n}\frac{\left(H_{\left(i,j+1\right)}^{n+1}-H_{\left(i,j-1\right)}^{n}\right)}{\Delta \tau } \end{array}$$

(15) $$E_{\left(i,j\right)}S_{\left(i,j-1\right)}^{n+2}+F_{\left(i,j\right)}S_{\left(i,j\right)}^{n+2}+G_{\left(i,j\right)}S_{\left(i,j+1\right)}^{n+2}=I_{\left(i,j\right)}$$

(16) $$E_{\left(i,j\right)}=-\frac{{\left(H_{\left(i,j\right)}^{n+1}\right)}^{3}}{{\left(\Delta \theta \right)}^{2}}-\frac{\Lambda_{J}H_{\left(i,j\right)}^{n+1}}{4P_{\left(i,j\right)}^{n+1}\Delta \theta }+3{\left(H_{\left(i,j\right)}^{n+1}\right)}^{2}\frac{H_{\left(i,j+1\right)}^{n+1}-H_{\left(i,j-1\right)}^{n+1}}{{\left(2\Delta \theta \right)}^{2}}$$

(17) $$F_{\left(i,j\right)}=\frac{2{\left(H_{\left(i,j\right)}^{n+1}\right)}^{3}}{{\left(\Delta \theta \right)}^{2}}+\frac{\Lambda_{J}H_{\left(i,j\right)}^{n+1}}{P_{\left(i,j\right)}^{n+1}\Delta \tau }$$

(18) $$G_{\left(i,j\right)}=-\frac{{\left(H_{\left(i,j\right)}^{n+1}\right)}^{3}}{{\left(\Delta \theta \right)}^{2}}+\frac{\Lambda_{J}H_{\left(i,j\right)}^{n+1}}{4P_{\left(i,j\right)}^{n+1}\Delta \theta }-3{\left(H_{\left(i,j\right)}^{n+1}\right)}^{2}\frac{H_{\left(i,j+1\right)}^{n+1}-H_{\left(i,j-1\right)}^{n+1}}{{\left(2\Delta \theta \right)}^{2}}$$

(19) $$\begin{array}{l} I_{\left(i,j\right)}=Q\delta_{k}+3{\left(H_{\left(i,j\right)}^{n+1}\right)}^{2}\frac{\left(H_{\left(i,j+1\right)}^{n+1}-H_{\left(i,j-1\right)}^{n+1}\right)\left(S_{\left(i,j+1\right)}^{n+1}-S_{\left(i,j-1\right)}^{n+1}\right)}{{\left(2\Delta Z\right)}^{2}}+\\ {\left(H_{\left(i,j\right)}^{n+1}\right)}^{3}\frac{\left(S_{\left(i+1,j\right)}^{n+1}-2S_{\left(i,j\right)}^{n+1}+S_{\left(i-1,j\right)}^{n+1}\right)}{{\left(\Delta Z\right)}^{2}}-\Lambda_{J}P_{\left(i,j\right)}^{n+1}\frac{H_{\left(i,j+1\right)}^{n+1}-H_{\left(i,j-1\right)}^{n+1}}{2\Delta \theta }-\\ 2\Lambda_{J}P_{\left(i,j\right)}^{n+1}\frac{H_{\left(i,j\right)}^{n+2}-H_{\left(i,j\right)}^{n+1}}{\Delta \tau }+\frac{\Lambda_{J}H_{\left(i,j\right)}^{n+1}S_{\left(i,j\right)}^{n+1}}{P_{\left(i,j\right)}^{n+1}\Delta \tau } \end{array}$$

The increment in the marching direction Z is carried out at the time step of n + 1, and the increment in the marching direction θ is carried out at the time step of n + 2. The Equation (10) and Equation (15) can be solved by the Thomas method, Then, the pressure distribution of the aerostatic journal bearing is obtained. Base on the load formulas given by Rowe [30], the air film force (i.e., the restoring force of the journal bearing) of the journal bearing in x and y direction is given by

(20) $$f_{bjx}=P_{s}L_{j}^{2}\int_{0}^{\frac{L}{L_{j}}}\int_{0}^{\frac{2\pi R}{L_{j}}}P\left(Z,\theta ,\tau \right)\cos\left(\alpha_{\left(i\right)}\right)d\theta dZ$$

(21) $$f_{bjy}=P_{s}L_{j}^{2}\int_{0}^{\frac{L}{L_{j}}}\int_{0}^{\frac{2\pi R}{L_{j}}}P\left(Z,\theta ,\tau \right)\sin\left(\alpha_{\left(i\right)}\right)d\theta dZ$$

And the torque of the air film force (i.e., the restoring torque of the journal bearing) on the barycenter of the spindle shaft respect to the x axis and y axis is given by

(22) $$t_{bjx}=P_{s}L_{j}^{2}L_{s}\frac{i-i_{d}}{M_{s}}\int_{0}^{\frac{L}{L_{j}}}\int_{0}^{\frac{2\pi R}{L_{j}}}P\left(Z,\theta ,\tau \right)\sin\left(\alpha_{\left(i\right)}\right)d\theta dZ$$

(23) $$t_{bjy}=P_{s}L_{j}^{2}L_{s}\frac{i-i_{d}}{M_{s}}\int_{0}^{\frac{L}{L_{j}}}\int_{0}^{\frac{2\pi R}{L_{j}}}P\left(Z,\theta ,\tau \right)\cos\left(\alpha_{\left(i\right)}\right)d\theta dZ$$

### 2.2. Modeling of Aerostatic Thrust Bearing

The transient pressure distribution of aerostatic thrust bearing can be modeled by the transient Reynolds equation in cylindrical coordinate.

(24) $$\frac{1}{r}\frac{\partial }{\partial r}\left(rph^{3}\frac{\partial p}{\partial r}\right)+\frac{1}{r^{2}}\frac{\partial }{\partial \theta }\left(ph^{3}\frac{\partial p}{\partial \theta }\right)+\frac{12\mu P_{a}}{\rho_{a}}\rho \tilde{v}\delta_{k}=\frac{6}{r}\frac{\partial }{\partial \theta }\left(phV_{0}\right)+12\mu \frac{\partial \left(ph\right)}{\partial t}$$

where θ is the circumferential coordinates of journal bearing, r is the radial coordinate of journal bearing, $V_{0}$ is the surface velocity of the shaft.

The dimensionless transient Reynolds equation is given by

(25) $$\frac{\partial }{\partial \theta }\left(H^{3}\frac{\partial P^{2}}{\partial \theta }\right)+R_{r}\frac{\partial }{\partial R_{r}}\left(R_{r}H^{3}\frac{\partial P^{2}}{\partial R_{r}}\right)+Q\delta_{k}=\Lambda_{T}\frac{\partial \left(PH\right)}{\partial \theta }+2\Lambda_{T}\frac{\partial \left(PH\right)}{\partial \tau }$$

The dimensionless parameters are defined as follows.

$$P=\frac{p}{P_{s}},H=\frac{h}{C_{r}},R_{r}=\frac{r}{L_{t}},Q=\frac{24\mu r^{2}P_{a}}{C_{r}^{3}P_{s}^{2}\rho_{a}}\rho \tilde{v},\tau =\omega t,\Lambda_{T}=\frac{12\mu \omega r^{2}}{P_{s}C_{r}^{2}}$$

where $L_{t}$ is the characteristic length of journal bearing, and $\Lambda_{T}$ is the bearing number of the thrust bearing.

**Figure 4:**
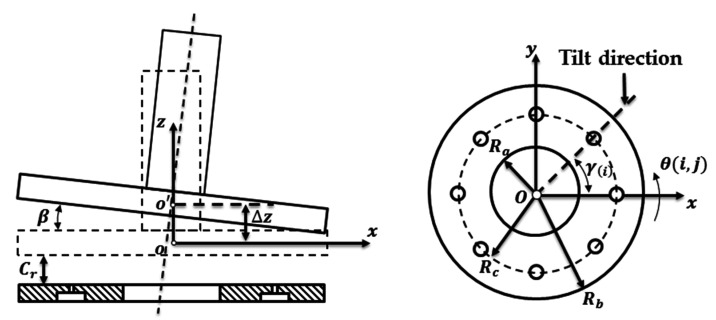
The tilt of the shaft at the thrust bearing.

The tilt motion of the shaft is considered to have influence on the air film thickness of the thrust bearing while the influence of the eccentricity is neglected. The tilt motion at the thrust bearing is shown in Figure 4, and the distribution of the air film thickness at the thrust bearing can be expressed as

(26) $$h_{\left(i,j\right)}=C_{r}-\sin\left(\beta \right)\sin\left(\theta_{\left(i,j\right)}-\gamma_{\left(i\right)}+\frac{\pi }{2}\right)\cdot \left(\frac{i}{M_{t}}\left(R_{b}-R_{a}\right)+R_{a}\right)+\Delta z$$

where $\theta_{\left(i,j\right)}$ is the angle of air film at node i and j, $\gamma_{\left(i\right)}$ is the angle between the tile direction and the positive direction of x at the node i, i is the node number along the radial direction, $R_{a}$ is the inner diameter of the thrust bearing, $R_{b}$ is the out diameter of the thrust bearing, $M_{t}$ is the number of thrust bearing nodes along the radial direction, Δz is the shaft displacement in the axial direction. 

**Figure 5:**
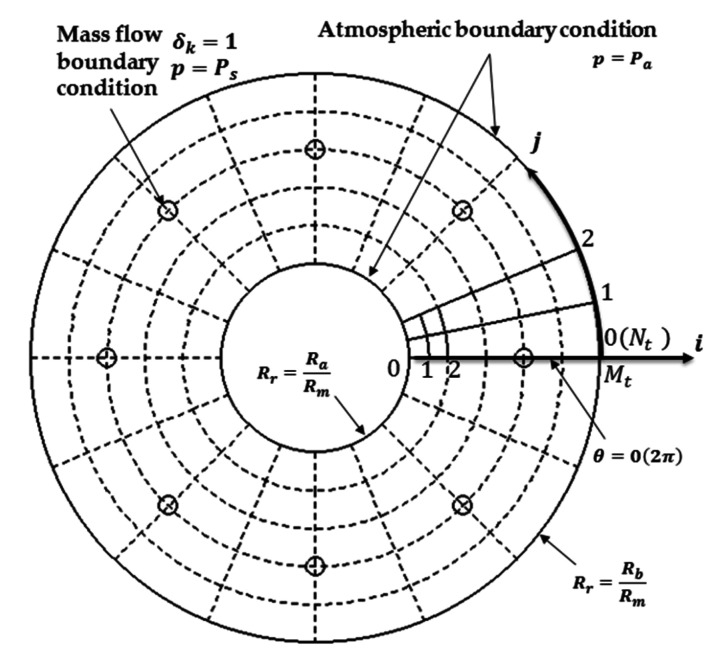
The computational domain of the thrust bearing.

As shown in Figure 5, the computational domain of the thrust bearing film is meshed into $\theta \left(0:N_{t}\right)$ and $R_{r}\left(0:M_{t}\right)$, the Periodic boundary condition, the Atmospheric boundary condition and the Mass flow boundary condition are also defined.

Adopt the same treatment as the journal bearing, the ADI format of the equation obtained from thrust bearing can be expressed as

(27) $$A_{\left(i,j\right)}S_{\left(i-1,j\right)}^{n+1}+B_{\left(i,j\right)}S_{\left(i,j\right)}^{n+1}+C_{\left(i,j\right)}S_{\left(i+1,j\right)}^{n+1}=D_{\left(i,j\right)}$$

(28) $$A_{\left(i,j\right)}=-\frac{R_{r}{}_{{}_{\left(i,j\right)}}^{2}{\left(H_{\left(i,j\right)}^{n}\right)}^{3}}{{\left(\Delta R_{r}\right)}^{2}}+R_{r}{}_{{}_{\left(i,j\right)}}^{2}3{\left(H_{\left(i,j\right)}^{n}\right)}^{2}\frac{H_{\left(i+1,j\right)}^{n}-H_{\left(i-1,j\right)}^{n}}{{\left(2\Delta R_{r}\right)}^{2}}+\frac{R_{r}{}_{{}_{\left(i,j\right)}}{\left(H_{\left(i,j\right)}^{n}\right)}^{3}}{2\Delta R_{r}}$$

(29) $$B_{\left(i,j\right)}=\frac{2R_{r}{}_{{}_{\left(i,j\right)}}^{2}{\left(H_{\left(i,j\right)}^{n}\right)}^{3}}{{\left(\Delta R_{r}\right)}^{2}}+\frac{\Lambda_{T}H_{\left(i,j\right)}^{n}}{P_{\left(i,j\right)}^{n}\Delta \tau }$$

(30) $$C_{\left(i,j\right)}=-\frac{R_{r}{}_{{}_{\left(i,j\right)}}^{2}{\left(H_{\left(i,j\right)}^{n}\right)}^{3}}{{\left(\Delta R_{r}\right)}^{2}}-R_{r}{}_{{}_{\left(i,j\right)}}^{2}3{\left(H_{\left(i,j\right)}^{n}\right)}^{2}\frac{H_{\left(i+1,j\right)}^{n}-H_{\left(i-1,j\right)}^{n}}{{\left(2\Delta R_{r}\right)}^{2}}-\frac{R_{r}{}_{{}_{\left(i,j\right)}}{\left(H_{\left(i,j\right)}^{n}\right)}^{3}}{2\Delta R_{r}}$$

(31) $$\begin{array}{l} D_{\left(i,j\right)}=Q\delta_{k}+3{\left(H_{\left(i,j\right)}^{n}\right)}^{2}\frac{\left(H_{\left(i,j+1\right)}^{n}-H_{\left(i,j-1\right)}^{n}\right)\left(S_{\left(i,j+1\right)}^{n}-S_{\left(i,j-1\right)}^{n}\right)}{{\left(2\Delta \theta \right)}^{2}}+\\ {\left(H_{\left(i,j\right)}^{n}\right)}^{3}\frac{\left(S_{\left(i,j+1\right)}^{n}-2S_{\left(i,j\right)}^{n}+S_{\left(i,j-1\right)}^{n}\right)}{{\left(\Delta \theta \right)}^{2}}-\frac{\Lambda_{T}H_{\left(i,j\right)}^{n}}{2P_{\left(i,j\right)}^{n}}\frac{\left(S_{\left(i,j+1\right)}^{n}-S_{\left(i,j-1\right)}^{n}\right)}{2\Delta \theta }\\ -\Lambda_{T}P_{\left(i,j\right)}^{n}\frac{\left(H_{\left(i,j+1\right)}^{n}-H_{\left(i,j-1\right)}^{n}\right)}{2\Delta \theta }-\frac{\Lambda_{T}H_{\left(i,j\right)}^{n}S_{\left(i,j\right)}^{n}}{P_{\left(i,j\right)}^{n}\Delta \tau }-2\Lambda_{T}P_{\left(i,j\right)}^{n}\frac{\left(H_{\left(i,j+1\right)}^{n+1}-H_{\left(i,j-1\right)}^{n}\right)}{\Delta \tau } \end{array}$$

(32) $$E_{\left(i,j\right)}S_{\left(i,j-1\right)}^{n+2}+F_{\left(i,j\right)}S_{\left(i,j\right)}^{n+2}+G_{\left(i,j\right)}S_{\left(i,j+1\right)}^{n+2}=I_{\left(i,j\right)}$$

(33) $$E_{\left(i,j\right)}=-\frac{{\left(H_{\left(i,j\right)}^{n+1}\right)}^{3}}{{\left(\Delta \theta \right)}^{2}}-\frac{\Lambda_{T}H_{\left(i,j\right)}^{n+1}}{4P_{\left(i,j\right)}^{n+1}\Delta \theta }+3{\left(H_{\left(i,j\right)}^{n+1}\right)}^{2}\frac{H_{\left(i,j+1\right)}^{n+1}-H_{\left(i,j-1\right)}^{n+1}}{{\left(2\Delta \theta \right)}^{2}}$$

(34) $$F_{\left(i,j\right)}=\frac{2{\left(H_{\left(i,j\right)}^{n+1}\right)}^{3}}{{\left(\Delta \theta \right)}^{2}}+\frac{\Lambda_{T}H_{\left(i,j\right)}^{n+1}}{P_{\left(i,j\right)}^{n+1}\Delta \tau }$$

(35) $$G_{\left(i,j\right)}=-\frac{{\left(H_{\left(i,j\right)}^{n+1}\right)}^{3}}{{\left(\Delta \theta \right)}^{2}}+\frac{\Lambda_{T}H_{\left(i,j\right)}^{n+1}}{4P_{\left(i,j\right)}^{n+1}\Delta \theta }-\frac{R_{r_{\left(i,j\right)}}{\left(H_{\left(i,j\right)}^{n+1}\right)}^{3}}{2\Delta \theta }-3{\left(H_{\left(i,j\right)}^{n+1}\right)}^{2}\frac{H_{\left(i,j+1\right)}^{n+1}-H_{\left(i,j-1\right)}^{n+1}}{{\left(2\Delta \theta \right)}^{2}}$$

(36) $$\begin{array}{l} I_{\left(i,j\right)}=Q\delta_{k}+3R_{r}{}_{{}_{\left(i,j\right)}}^{2}{\left(H_{\left(i,j\right)}^{n+1}\right)}^{2}\frac{\left(H_{\left(i,j+1\right)}^{n+1}-H_{\left(i,j-1\right)}^{n+1}\right)\left(S_{\left(i,j+1\right)}^{n+1}-S_{\left(i,j-1\right)}^{n+1}\right)}{{\left(2\Delta R_{r}\right)}^{2}}+\\ R_{r}{}_{{}_{\left(i,j\right)}}^{2}{\left(H_{\left(i,j\right)}^{n+1}\right)}^{3}\frac{\left(S_{\left(i+1,j\right)}^{n+1}-2S_{\left(i,j\right)}^{n+1}+S_{\left(i-1,j\right)}^{n+1}\right)}{{\left(\Delta R_{r}\right)}^{2}}-\Lambda_{T}P_{\left(i,j\right)}^{n+1}\frac{H_{\left(i,j+1\right)}^{n+1}-H_{\left(i,j-1\right)}^{n+1}}{2\Delta \theta }-\\ 2\Lambda_{T}P_{\left(i,j\right)}^{n+1}\frac{H_{\left(i,j\right)}^{n+2}-H_{\left(i,j\right)}^{n+1}}{\Delta \tau }+\frac{\Lambda_{T}H_{\left(i,j\right)}^{n+1}S_{\left(i,j\right)}^{n+1}}{P_{\left(i,j\right)}^{n+1}\Delta \tau } \end{array}$$

By solving the Equation (27) and (32) using Thomas method, the pressure distribution of the thrust bearing can be obtained. The air film force (i.e., the restoring force of the thrust bearing) of the thrust bearing in z direction is given by

(37) $$f_{btz}=P_{s}L_{t}^{2}\int_{0}^{\frac{R_{b}-R_{a}}{L_{t}}}\int_{0}^{2\pi }P\left(R_{r},\theta ,\tau \right)\cos\left(\beta \right)R_{r}d\theta dR_{r}$$

And the torque of the air film force (i.e., the restoring torque of the thrust bearing) of the thrust bearing respect to the x axis and y axis is given by

(38) $$t_{btx}=P_{s}L_{t}^{2}\left(R_{a}+\frac{i\left(R_{b}-R_{a}\right)}{M_{r}}\right)\int_{0}^{\frac{R_{b}-R_{a}}{L_{t}}}\int_{0}^{2\pi }P\left(R_{r},\theta ,\tau \right)\cos\left(\beta \right)\sin\left(\alpha_{\left(i\right)}\right)R_{r}d\theta dR_{r}$$

(39) $$t_{bty}=P_{s}L_{t}^{2}\left(R_{a}+\frac{i\left(R_{b}-R_{a}\right)}{M_{r}}\right)\int_{0}^{\frac{R_{b}-R_{a}}{L_{t}}}\int_{0}^{2\pi }P\left(R_{r},\theta ,\tau \right)\cos\left(\beta \right)\cos\left(\alpha_{\left(i\right)}\right)R_{r}d\theta dR_{r}$$

### 2.3. Modeling of PMSM

Ideally, the magnetic force of PMSM is symmetric. However, the UMF will come out when the rotor eccentricity happens or the structure of the PMSM is not symmetric. Kawase et al. [31] used 3-D finite element method to analyze the UMF of the PMSM, and it is found that the axial component of the UMF is relatively small compared to other two components. Here, we assume the structure of the PMSM is symmetric and the axial component of the UMF is negligible. According to Maxwell stress tensor method, the 2D magnetic forces in the radial and tangential direction can be expressed as follows [32].

(40) $$f_{r}=\int_{0}^{2\pi }\frac{1}{2\mu_{0}}\left(B_{r}^{2}-B_{\theta }^{2}\right)rd\theta$$

(41) $$f_{\theta }=\int_{0}^{2\pi }\frac{B_{r}B_{\theta }}{\mu_{0}}rd\theta$$

where $B_{r}$ is the radial flux density, $B_{\theta }$ is the tangential flux density, $\mu_{0}$ is the permeability of the air.

**Figure 6:**
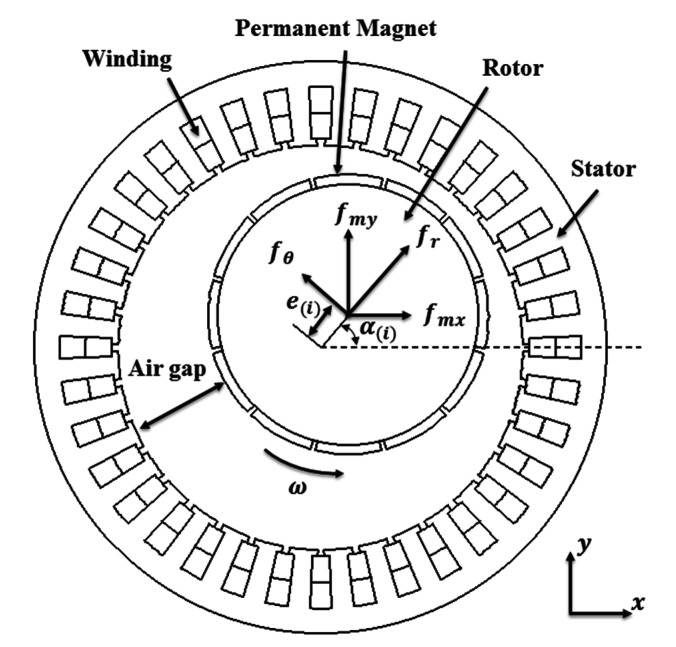
Schematic structure of the motor [4].

Figure 6 shows the schematic structure of motor with eccentric rotor [4], and the 2-D magnetic force in cartesian coordinates can be expressed as

(42) $$f_{mx_{\left(i\right)}}=f_{r_{\left(i\right)}}\cos\left(\alpha_{\left(i\right)}\right)+f_{\theta }sin\left(\alpha_{\left(i\right)}\right)$$

(43) $$f_{mx_{\left(i\right)}}=f_{r_{\left(i\right)}}\sin\left(\alpha_{\left(i\right)}\right)+f_{\theta_{\left(i\right)}}\cos\left(\alpha_{\left(i\right)}\right)$$

**Figure 7:**
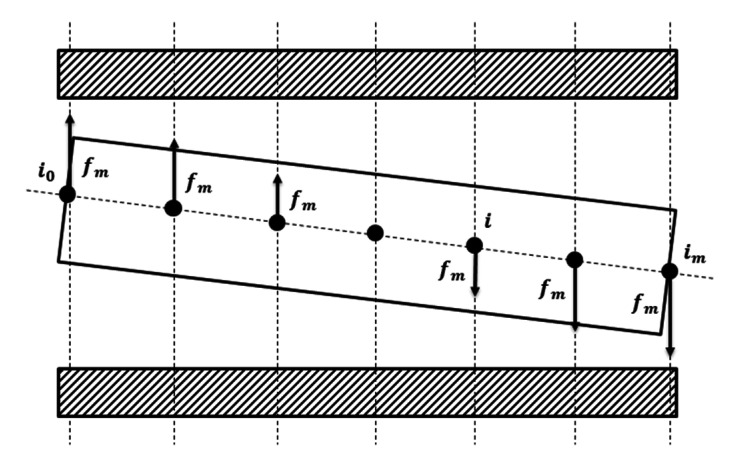
The simplified PMSM model using multiple slice method.

To simulate the 3-D state of the PMSM, the multiple slice method is employed. As shown in Figure 7, the magnetic force of the 3-D PMSM can be expressed as

(44) $$f_{mx}=\sum_{i=i_{0}}^{i=i_{m}}f_{mx_{\left(i\right)}}$$

(45) $$f_{my}=\sum_{i=i_{0}}^{i=i_{m}}f_{my_{\left(i\right)}}$$

And the magnetic torque of the 3-D PMSM is given by

(46) $$t_{mx}=\sum_{i=i_{0}}^{i=i_{m}}f_{my_{\left(i\right)}}\frac{i-i_{b}}{M_{s}}L_{s}$$

(47) $$t_{my}=\sum_{i=i_{0}}^{i=i_{m}}f_{mx_{\left(i\right)}}\frac{i-i_{b}}{M_{s}}L_{s}$$

### 2.4. Dynamics Modeling of ABMS

The spindle shaft is regarded as a rigid rotor in the model, and the spindle shaft is considered to move with 5-DOF. The dimensionless acceleration, velocity and the attitude in the space can be calculated by

(48) $$\left\{\ddot{\delta }\right\}=\frac{d^{2}\left\{\delta \right\}}{d\tau^{2}}=\left\{A\right\}$$

(49) $$\left\{\dot{\delta }\right\}=\left\{{\dot{\delta }}_{0}\right\}+\left\{\ddot{\delta }\right\}\Delta \tau$$

(50) $$\left\{\delta \right\}=\left\{\delta_{0}\right\}+\left\{\dot{\delta }\right\}\Delta \tau +\frac{1}{2}\left\{\ddot{\delta }\right\}{\left(\Delta \tau \right)}^{2}$$

(51) $$\left\{A\right\}=\left\{\begin{array}{l} \frac{F_{eX}-F_{bX}+F_{mX}}{M}\\ \frac{F_{eY}-F_{bY}+F_{mY}}{M}\\ \frac{F_{eZ}-F_{bZ}+F_{mZ}}{M}\\ \frac{T_{eX}-T_{bX}+T_{mX}+\left(I_{z}-I_{x}\right){\dot{\Theta }}_{Y}{\dot{\Theta }}_{Z}}{I_{X}}\\ \frac{T_{eY}-T_{bY}+T_{mY}+\left(I_{y}-I_{z}\right){\dot{\Theta }}_{Z}{\dot{\Theta }}_{X}}{I_{Y}} \end{array}\right\}$$

(52) $$\left\{\delta \right\}=\left\{\begin{array}{l} X\\ Y\\ Z\\ \Theta_{X}\\ \Theta_{Y} \end{array}\right\}$$

where the dimensionless parameters are given as follows.

$$\begin{array}{l} X=\frac{x}{C_{r}},Y=\frac{y}{C_{r}},Z=\frac{z}{C_{r}},F_{ex}=\frac{f_{ex}}{P_{s}L_{r}^{2}},F_{ey}=\frac{f_{ey}}{P_{s}L_{r}^{2}},F_{ex}=\frac{f_{ez}}{P_{s}L_{r}^{2}},F_{bx}=\frac{f_{bx}}{P_{s}L_{r}^{2}},\\ F_{by}=\frac{f_{by}}{P_{s}L_{r}^{2}},F_{bz}=\frac{f_{bz}}{P_{s}L_{r}^{2}},F_{mx}=\frac{f_{mx}}{P_{s}L_{r}^{2}},F_{my}=\frac{f_{my}}{P_{s}L_{r}^{2}},T_{ex}=\frac{t_{ex}}{P_{s}L_{r}^{2}L_{s}},T_{ey}=\frac{t_{ey}}{P_{s}L_{r}^{2}L_{s}},\\ T_{bx}=\frac{t_{bjx}+t_{btx}}{P_{s}L_{r}^{2}L_{s}},T_{by}=\frac{t_{bjy}+t_{bty}}{P_{s}L_{r}^{2}L_{s}},T_{mx}=\frac{t_{mx}}{P_{s}L_{r}^{2}L_{s}},T_{my}=\frac{t_{my}}{P_{s}L_{r}^{2}L_{s}},\\ M=\frac{mC_{r}\omega^{2}}{P_{s}L_{r}^{2}},I_{x}=\frac{i_{x}\omega^{2}}{P_{s}L_{r}^{2}L_{s}},I_{y}=\frac{i_{y}\omega^{2}}{P_{s}L_{r}^{2}L_{s}},I_{z}=\frac{i_{z}\omega^{2}}{P_{s}L_{r}^{2}L_{s}} \end{array}$$

where $F_{ex},F_{ey},F_{ez}$ are the dimensionless external force applied on the spindle shaft in x,y,z direction, $F_{bx},F_{by},F_{bz}$ are the dimensionless air film force in x,y,z direction, $F_{mx},F_{my}$ are the dimensionless UMF in x,y direction, $T_{ex},T_{ey}$ are the dimensionless external torque with respect to x,y axis, $T_{bx},T_{by}$ are the dimensionless air film torque with respect to x,y axis, $T_{mx},T_{my}$ are the dimensionless magnetic torque with respect to x,y axis, M is the dimensionless mass of the spindle shaft, $I_{x},I_{y},I_{z}$ are the rotational inertia of the spindle shaft with respect to x,y,z axis, X,Y,Z are the dimensionless displacement in x,y,z direction, $\Theta_{X},\Theta_{Y}$ are the rotation angle of the spindle shaft with respect to x,y axis.

**Figure 8:**
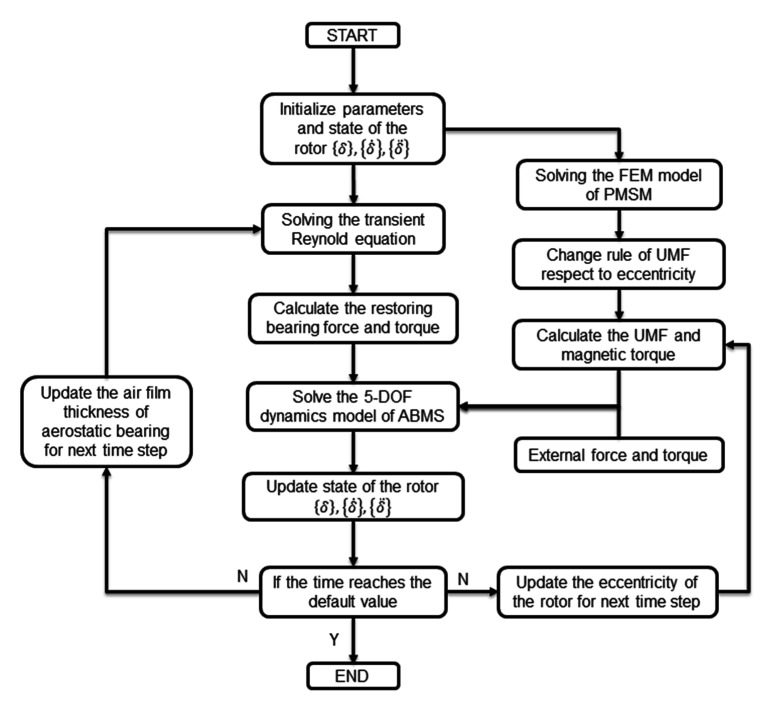
Flow chat for calculating the 5-DOF dynamics model of ABMS.

The spindle shaft is still at the initial condition, thus $\left\{\dot{\delta }\right\}=\left\{0\right\}$ at the initial time. The flow chat for calculating the 5-DOF dynamics model of the ABMS is shown in Figure 8. 

## 3. Numerical Simulation

### 3.1. Detailed Parameter

A case study is conducted to verify the effectiveness of the dynamics model of the ABMS. Table 1 lists the detailed parameters of the simulated model. 

### 3.2. Numerical Result

The model selects the same motor parameter as that in reference [4], and the variation trend of UMF has been given as follows.

(53) $$f_{mx}=\{\begin{array}{c} \begin{array}{cc} \left[\left(-4.4\varepsilon^{2}+7.4\varepsilon +0.3\right)\cdot \sin\left(\frac{2\pi \omega }{60}t\right)+\left(9.4\varepsilon^{2}-3.1\varepsilon +3.8\right)\right]\cdot \sin\theta_{r} & \left(\varepsilon \leq 0.5\right) \end{array}\\ \begin{array}{cc} \left[\left(-4.4\varepsilon^{2}+7.4\varepsilon +0.3\right)\cdot \sin\left(\frac{2\pi \omega }{60}t\right)+\left(7.0\varepsilon +1.1\right)\right]\cdot \sin\theta_{r} & \left(\varepsilon >0.5\right) \end{array} \end{array}$$

(54) $$f_{my}=\{\begin{array}{c} \begin{array}{cc} \left[\left(-4.4\varepsilon^{2}+7.4\varepsilon +0.3\right)\cdot \sin\left(\frac{2\pi \omega }{60}t\right)+\left(9.4\varepsilon^{2}-3.1\varepsilon +3.8\right)\right]\cdot \cos\theta_{r} & \left(\varepsilon \leq 0.5\right) \end{array}\\ \begin{array}{cc} \left[\left(-4.4\varepsilon^{2}+7.4\varepsilon +0.3\right)\cdot \sin\left(\frac{2\pi \omega }{60}t\right)+\left(7.0\varepsilon +1.1\right)\right]\cdot \cos\theta_{r} & \left(\varepsilon >0.5\right) \end{array} \end{array}$$

(55) $$\theta_{r}=\left(9.3\varepsilon^{3}-13.4\varepsilon^{2}+1.8\varepsilon +2.8\right)\cdot \sin\left(\frac{2\pi \omega }{60}t\right)$$

**Figure 9:**
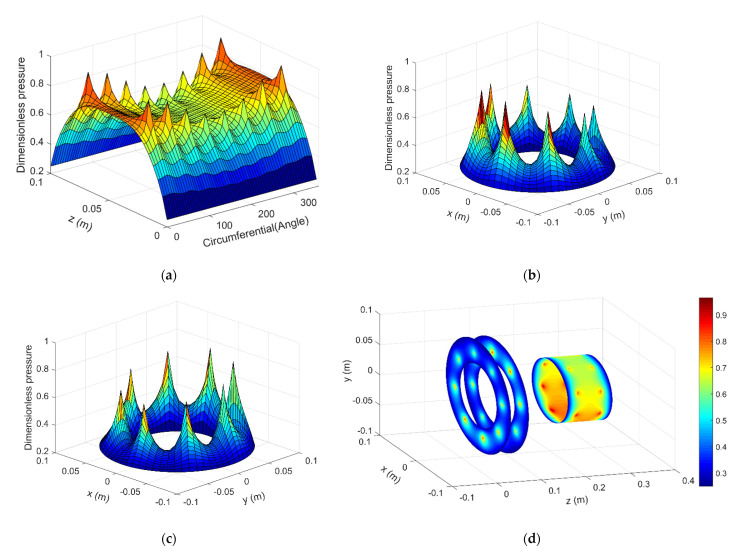
Pressure distribution of the aerostatic bearings with 400 N external force: (**a**) Journal bearing (**b**) Front thrust bearing (**c**) Rear thrust bearing (**d**) Integrated vision.

Two cases are calculated, i.e., the dynamics model with 400 N and 50 N external force. In the first case, 400 N external force is applied on the shaft end in x direction, the default dimensionless time is set to be 100. Figure 9a shows the air pressure distribution of the aerostatic journal bearing, Figure 9b and c show the air pressure distribution of the front and rear aerostatic journal bearing respectively, And Figure 9d shows the integrated air pressure distribution vision of aerostatic bearing with 400 N external force at dimensionless time 100.

**Figure 10:**
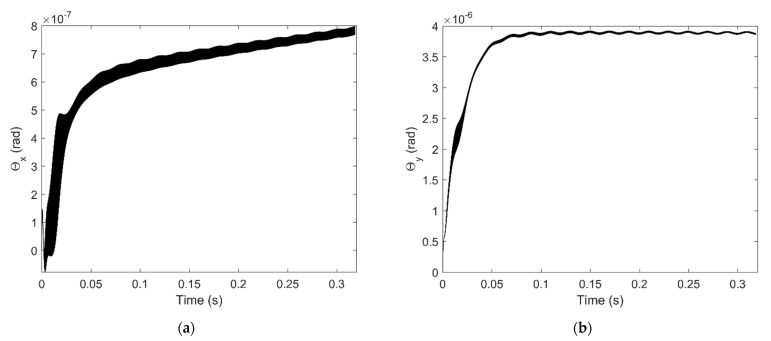
Tilt angle of the spindle shaft with 400 N external force: (**a**) Tilt angle versus x-axis (**b**) Tilt angle versus y-axis.

The result shows that when applies the external load on the shaft end, the spindle shaft can have a certain tilt angle and it affects the air pressure distribution of the aerostatic bearings obviously, which means the restoring force and torque of the air film applied on the spindle shaft are also changed. The tilt angle of the spindle shaft versus x axis and y axis is shown in Figure 10a,b, the tilt angle is converged and varies periodically with a certain amplitude, the extent of the tilt angle versus y axis is larger than the tilt angle versus x axis, this is mainly because the external force is applied in the x direction, thus the external torque is applied on y axis.

**Figure 11:**
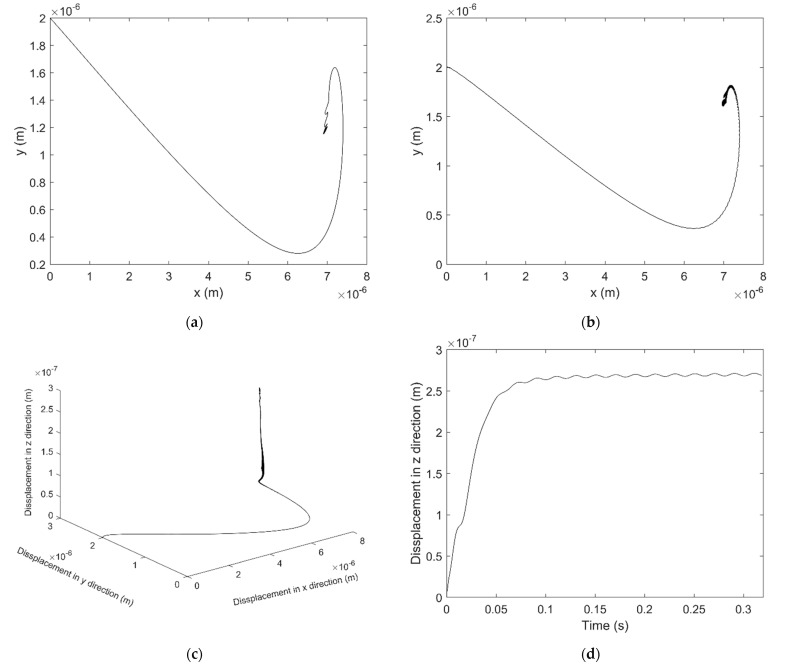
Motion trajectory of shaft with 400 N external force: (**a**) Position at the shaft barycenter in x-y plane (**b**) Position at the shaft end in x-y plane (**c**) Position at the shaft end in x-y-z coordinate (**d**) Position at the shaft end in z direction.

Figure 11a,b shows the motion trajectory of the shaft barycenter and the shaft end respectively. The result shows that the shaft acts stable with 400 N external force, its trajectory in x-y plane converged to a certain region. From a spatial perspective, the shaft end has left its initial position towards its balance position as shown in Figure 11c, and the displacement of the shaft end in z direction is shown in Figure 11d. The displacement in z direction is also converged and it varies periodically versus time.

**Figure 12:**
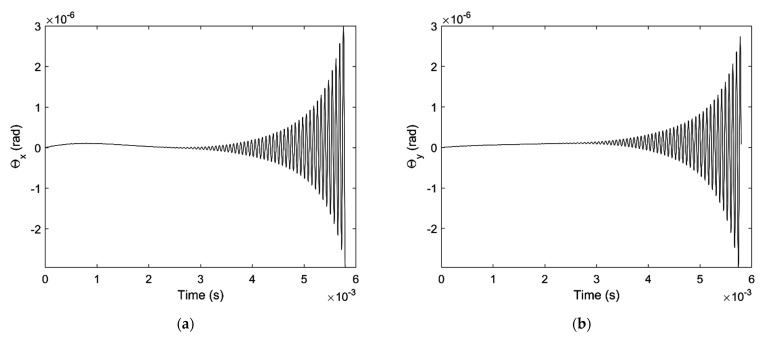
Tilt angle of the shaft with 50N external force: (**a**) Tilt angle versus x-axis (**b**) Tilt angle versus y-axis.

In the second case, 50 N external force is applied on the shaft end in x direction. The result is quite different with the first case, Figure 12a,b shows the tilt angle of the shaft versus x axis and y axis which are both diverged, which means the shaft is unstable under the current condition.

**Figure 13:**
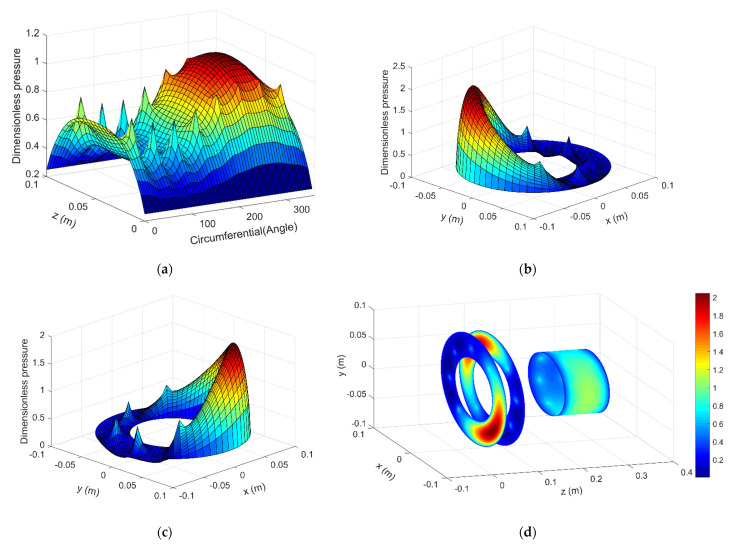
Pressure distribution of the aerostatic bearings with 50 N external force: (**a**) Journal bearing (**b**) Front thrust bearing (**c**) Rear thrust bearing (**d**) Integrated vision.

Figure 13a shows the corresponding air pressure distribution of the aerostatic journal bearing, Figure 13b,c show the air pressure distribution of the front and rear aerostatic journal bearing respectively, Figure 13d shows the integrated air film pressure distribution vision of aero-static bearing with 50 N external force at dimensionless time 100. According to the result, the air film pressure distribution of the aerostatic bearing becomes quite uneven, both the aerostatic journal bearing and the aerostatic thrust bearing show severe aerodynamic effect. 

**Figure 14:**
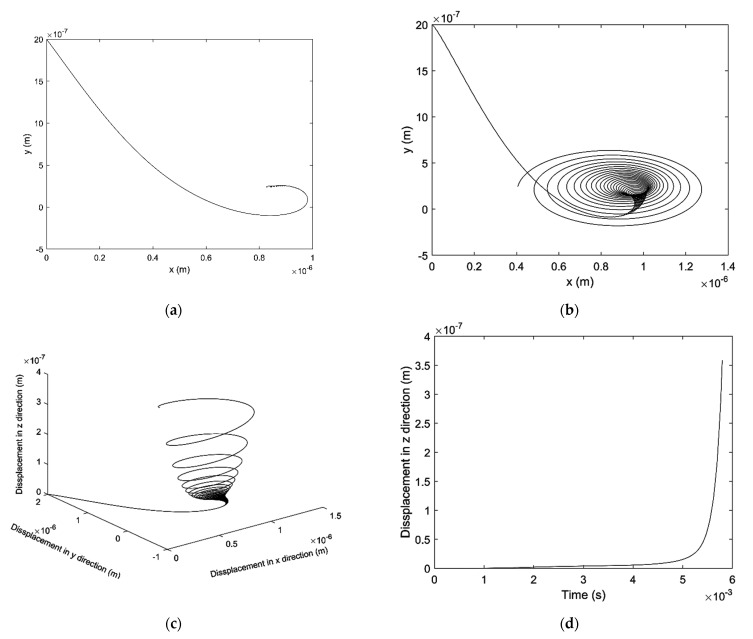
Motion trajectory of the aerostatic bearings with 50 N external force: (**a**) Journal bearing (**b**) Front thrust bearing (**c**) Rear thrust bearing (**d**) Integrated vision.

Figure 14a,b shows the motion trajectory of the shaft barycenter and the shaft end respectively. The result shows that the trajectory of shaft barycenter does not show the divergent trend while the trajectory of shaft end does, which means that the spindle shaft is at conical unstable state. It can be observed clearer in a spatial perspective as shown in Figure 14c, the trajectory of the shaft end moves as a spiral path. The displacement of the shaft end in the axial direction is also divergent as shown in Figure 14d. 

## 4. Discussion

In the motion process of the spindle shaft, the external force applied on the shaft end produce the torque on the shaft, resulting in the tilt motion of the shaft, then the distribution of the motor flux and the air film pressure is changed. Under the multi-field coupling effect of the external force, the air film force and the magnetic force, the shaft may finally stabilize in a certain spatial attitude or become unstable and even hit the bearing sleeve. 

In this paper, a 5-DOF dynamics model of the aerostatic spindle was implemented using the restoring force method, while previous literatures mainly used the dynamic coefficient method [23,33]. The dynamic coefficient method improves the calculation speed compared to the restoring force method, but the dynamic behavior of the shaft can be obtained directly by employing the restoring force method. The proposed 5-DOF dynamic model of aerostatic spindle has considered the influence of the tilt motion and axial displacement of the shaft, which is neglected in the 2-DOF model. In the 2-DOF model of aerostatic spindle only the external force, the restoring force of the air film and the unbalanced magnetic force of the motor are considered, while the external torque, the restoring torque of the air film and the magnetic torque of the motor are not considered. However, they are all considered and integrated in the 5-DOF model. The motion trajectory of the shaft center in the 2D plane characterizes the dynamic behavior of the shaft, but it cannot show the conical motion of the shaft with the external force and external torque, which is quite important because the shaft has the spatial motion with 5-DOF. 

In the simulation result, the dynamic behavior of the spindle shaft is stable with 400 N force and unstable with 50 N force. Similar phenomenon has been observed in the research of journal bearings, such as the research done by Khonsari et al. [34]. The stability boundary of the journal bearing varies with the change of the Sommerfeld Number, and the load on the bearing is one of the parameters that determine the value of the Sommerfeld Number. The stability boundary expands with the increase of the external load. To our knowledge, we think the reason behind dynamic behavior is probably the same as that of the journal bearings. That is, when the external load increase, the initial position locates in the unstable region under the low external load may locate in the stable region due to the expanding of the stability boundary. The Sommerfeld Number is also related to the rotation velocity [34]. Thus, the rotation velocity also affects the stability of the shaft, which is probably because that the change of the rotation velocity results in different aerodynamic effect and affects the stability. The critical value for distinguishing the stability of the shaft can be obtained in the 2-DOF model as the works done by Yang et al. [24]. It is sure that the critical value for the conical stability of the spindle shaft in the 5-DOF model also exists. It is meaningful to study the stability criterion of the shaft with 5-DOF. However, it will be much difficult to obtain the critical value in the 5-DOF model compared to that in the 2-DOF model. The 5-DOF model is much time costly to solve due to its high complexity and difficulty, thus, it will be further explored in the future research. 

The proposed model can be improved in somehow. The shaft is regarded is a rigid body rather than the flexible body, and the structural deformation is neglected, however, it may have potential influence on the dynamic behavior of the shaft. Laha et al. [35] investigated the stability of the rotor supported by air journal bearing considering the rotor flexibility, in which the object was 2-DOF model. If the shaft is considered as a flexible rotor in a 5-DOF model, extra calculations need to be done, and the improvement of the solving code is also required. Due to the limitation of the current experimental condition, the validation of the theoretical results will be conducted in the future work.

## 5. Conclusions

In this study, a 5-DOF dynamics model of the aerostatic spindle is established. The modeling method and the numerical solution is given. Through the numerical model, the transient pressure distribution of the air film as well as the motion trajectory of the spindle shaft is obtained as expected. The result shows the significant meaning of considering the influence of the shaft tilt on the air film pressure distribution, which further determines the restoring force and torque of the aerostatic bearing. Besides, the different conical stability behavior of the spindle shaft is shown under different external load through the model. 

## Figures and Tables

**Table 1:** The parameter of simulated ABMS.

Parameter (Unit)	Data
Length of shaft (m)	0.4
Diameter of journal bearing (m)	0.1
Length of journal bearing (m)	0.1
Inner diameter of thrust bearing (m)	0.1
Outer diameter of thrust bearing (m)	0.18
Bearing clearance (m)	1 × 10^−5^
Diameter of orifice (m)	0.0002
Row number of Orifice on journal bearing	2
Row number of Orifice on thrust bearing	1
Orifice number of each row	8
Ambient pressure (Pa)	101325
Supply pressure (Pa)	405300
Density of air (kg/m^3^)	1.204
Kinetic viscosity of air (Pa·s)	1.82 × 10^−5^
Specific heat ratio of air	1.401
Flow coefficient	0.8
Outer Rotor diameter (m)	0.1
Outer stator diameter (m)	0.15
Inner rotor diameter (m)	0.106
Number of slots	36
Number of poles	12
Motor effective length (m)	0.08
Rotational speed (r/min)	3000
Winding form of motor	Three-phase of double layer winding
External force (N)	50
	400

## Data Availability

No new data were created or analyzed in this study. Data sharing is not applicable to this article.
